# Effects of Mycotoxin-Sequestering Agents on Growth Performance and Nutrient Utilization of Growing Pigs Fed Deoxynivalenol-Contaminated Diets

**DOI:** 10.3390/life13101953

**Published:** 2023-09-23

**Authors:** Woong Bi Kwon, Seung Youp Shin, Yoon Soo Song, Changsu Kong, Beob Gyun Kim

**Affiliations:** 1 The Furst-McNess Company, Rockford, IL 61108, USA; woongbi.kwon@mcness.com; 2 Department of Animal Science and Technology, Konkuk University, Seoul 05029, Republic of Korea; shinsy02@konkuk.ac.kr (S.Y.S.); sang8sys@konkuk.ac.kr (Y.S.S.); 3 Department of Animal Science, Kyungpook National University, Sangju 37224, Republic of Korea; changsukong@knu.ac.kr

**Keywords:** deoxynivalenol, digestibility, feed intake, growing pigs, sequestering agent

## Abstract

The objective of this study was to investigate the effects of supplemental mycotoxin-sequestering agents on growth performance and nutrient utilization in growing pigs fed deoxynivalenol (DON)-contaminated diets. Twelve barrows with an initial body weight of 35.5 kg (standard deviation = 1.3) were assigned to six dietary treatments in a replicated 6 × 5 incomplete Latin square design. Five experimental diets consisted of an uncontaminated diet (PC), a DON-contaminated diet at 6.89 mg/kg (NC), NC + bentonite 0.5%, NC + yeast cell wall 0.5%, and NC + a mixture product 0.5% which consisted of enzymes, microorganisms, minerals, and plant extracts. Pigs had ad libitum access to the five diets. In the last group, the PC diet was restrictedly provided to pigs at the quantity of feed consumption of the NC group. Average daily gain, average daily feed intake, and gain:feed were not affected by supplemental mycotoxin-sequestering agents except for the mixed product that tended to improve (*p* = 0.064) gain:feed in pigs fed DON-contaminated diets. The apparent total tract digestibility (ATTD) of dry matter was not affected by DON contamination or by supplemental mycotoxin-sequestering agents, whereas the ATTD of Ca was decreased (*p* = 0.032) by supplemental yeast cell wall in pigs fed DON-contaminated diets. The ATTD of P was greater (*p* = 0.042) in pigs fed the NC diet compared with the pigs fed the restricted amount of the PC diet. In conclusion, bentonite and yeast cell wall did not affect growth performance of pigs fed DON-contaminated diets, but a supplemental mixed product consisting of enzymes, microorganisms, minerals, and plant extracts partially alleviated the negative effects of dietary DON on the gain:feed of pigs. Calcium digestibility was decreased by supplemental yeast cell wall in pigs fed DON-contaminated diets. Based on the present work, the use of a mixed product consisting of enzymes, microorganisms, minerals, and plant extracts is suggested, and the reduction of Ca digestibility by yeast cell wall needs to be considered in diet formulations.

## 1. Introduction

Mycotoxins are secondary toxic metabolites produced by several fungi on agricultural crops. Due to its frequent occurrence and toxicological concentrations, deoxynivalenol (DON) is one of the most prevalent mycotoxins generated by *Fusarium* fungus in cereal grains [1]. If a feed ingredient contaminated with DON is used in animal diets, DON in diets can cause negative effects in livestock including swine [2,3].

The negative effects of DON on growing pigs include reduced feed intake, decreased nutrient digestibility, and subsequently, reduced body weight gain [2,4,5,6]. The detrimental effects of DON on feed intake have been well demonstrated and summarized in a meta-analysis paper [7]. However, the influence of DON on nutrient digestibility is inconsistent among the literature. Jo et al. [5] reported that a DON-contaminated diet fed to pigs caused less ileal digestibility of amino acids compared with the control diet. On the other hand, Kong et al. [8] reported that dietary DON increased the apparent total tract digestibility (ATTD) of dry matter (DM) and crude protein in pigs.

To alleviate the negative effects of mycotoxins in diets, mycotoxin-sequestering agents are used in animal feeds [9,10]. Clay products including bentonite (BEN) and montmorillonite are known to have a high affinity for aflatoxins and are expected to restore the decreased performance of pigs caused by mycotoxins [11,12,13]. Yeast cell wall (YCW) products such as glucomannans and β-D-glucans have also showed a strong affinity for aflatoxins or zearalenone [9,14]. In addition to clay or yeast products, antioxidants, preservatives, and plant extracts are available to alleviate the negative effects of mycotoxins on the growth performance of pigs as a toxin adsorbent or a deactivator in forms of either a single or a mixed product. The effects of mycotoxin-sequestering agents on aflatoxins have been relatively consistent [9,11,15]. However, the efficacy of mycotoxin-sequestering agents on DON is often controversial [9,16,17], and thus, further information on DON-sequestering agents is necessary. Therefore, the objectives of this study were to determine the effects of DON on the growth performance and nutrient digestibility of pigs and to evaluate three products of toxin-sequestering agents including BEN, YCW, and a mixed product (MIX) containing enzymes, microorganisms, and plant extracts against the adverse effects of dietary DON in pigs. The mycotoxin-sequestering agents were hypothesized to potentially alleviate the negative effects of DON in diets fed to pigs. The information from the present work would be helpful for deciding which mycotoxin-sequestering agent to use for diet formulations.

## 2. Materials and Methods

The present experiment was reviewed and approved by the Institutional Animal Care and Use Committee of Konkuk University (KU15035).

### 2.1. Animals, Diets, and Experimental Design

Twelve barrows [Duroc × (Landrace × Yorkshire)] with an initial body weight (BW) of 35.5 ± 1.3 kg were used. Two sources of barley, one contaminated and one uncontaminated by DON, were obtained. The DON-contaminated barley contained 26 mg/kg of DON, whereas the uncontaminated barley contained 1 mg/kg of DON (Table 1).

**Figure 1:**
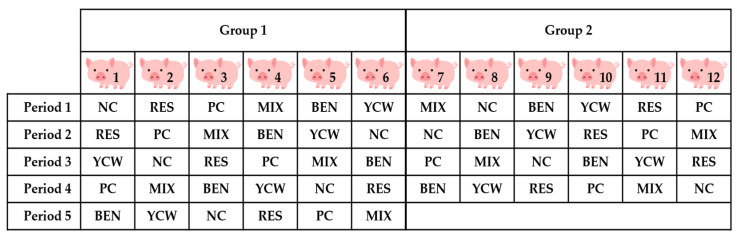
A replicated 6 × 5 incomplete Latin square design was employed using 12 animals, 6 experimental diets, and 5 periods balanced for potential carryover effects [19]. During period 5, only 6 pigs were used due to the lack of experimental diets, resulting in 9 observations for each treatment. PC = positive control of uncontaminated diet; RES = restricted feeding of an uncontaminated diet at the level of feed intake as in the negative control (NC) group; NC = deoxynivalenol-contaminated diet; BEN = NC + 0.5% bentonite; YCW = NC + 0.5% yeast cell wall; MIX = NC + 0.5% mixture product consisting of enzymes, microorganisms, minerals, and plant extracts.

The experimental diets were primarily based on corn, soybean meal, and barley (Table 2). The two sources of barley were used to prepare PC and NC diets. The five treatment groups were as follows: a positive control (PC) group fed an uncontaminated diet with no mycotoxin-sequestering agent; a negative control (NC) group fed a DON-contaminated diet with no mycotoxin-sequestering agent; NC + 0.5% BEN; NC + 0.5% YCW; and NC + 0.5% MIX containing enzymes, microorganisms, minerals, and plant extracts. In an additional group, pigs were fed the PC diet restrictedly (RES) at the level of voluntary feed intake in the NC group. Vitamins and minerals were included in the diets to meet or exceed the requirement estimates suggested by the NRC [18]. A replicated 6 × 5 incomplete Latin square design was employed using 12 animals, 6 experimental diets, and 5 periods (Figure 1) balanced for potential residual effects [19]. During period 5, only 6 pigs were used due to the lack of experimental diets, resulting in 9 observations for each treatment.

### 2.2. Feeding and Sample Collection

**Figure 2:**
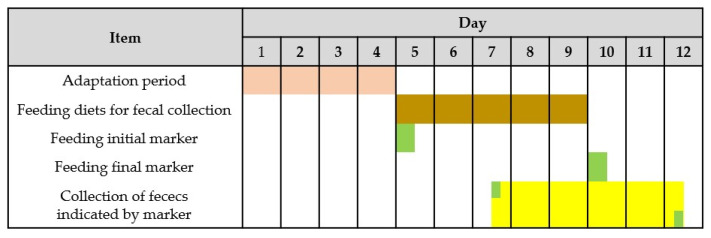
A period consisted of feeding for 4 days for adaptation (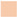) and feeding for 5 days for fecal collection (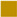). Chromium oxide was used as an indigestible marker for initiation and termination of fecal collection (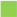). After feeding the initial and terminal marker to pigs in the morning of days 5 and 10, respectively, it took 2 to 3 days to the excretion of feces indicated by marker (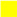).

All pigs were individually housed in metabolism crates equipped with a feeder and a nipple drinker. Individual BW was recorded at the beginning of each period. All pigs except those in the RES group had ad libitum access to the diets. Water was freely available at all times. Each period consisted of 4-day feeding for adaptation and 5-day feeding for fecal collection (Figure 2). During the 5-day feeding period for fecal collection, a total collection method was used to collect feces with the marker-to-marker procedure. Chromium oxide was used as an indigestible marker for the initiation and termination of fecal collection. After feeding the initial and terminal marker to pigs in the morning of days 5 and 10, respectively, it took 2 to 3 days to the excretion of feces indicated by the marker color. During the collection period, feed refusals were collected to calculate the amount of actual feed intake.

### 2.3. Chemical Analyses

Dry matter in diets and feces was determined, and Ca (method 935.13) and P (method 946.06) concentrations were also determined in diets and feces as suggested by the AOAC [20]. The concentrations of DON in ingredients and diets were determined using enzyme-linked immunosorbent assay kits (AgraQuant, Romer Labs Inc., Singapore) which had quantification ranges for analysis of DON from 250 to 5000 ng/mL.

### 2.4. Calculations and Statistical Analyses

The ATTD of nutrients in the experimental diets were calculated. Experimental data were analyzed using the MIXED procedure of SAS (SAS Inst. Inc., Cary, NC, USA). The model for the statistical analysis was as follows: Y*_ijkl_* = µ + trt*_i_* + replication*_j_* + period*_k_* (replication*_j_*) + animal*_l_* (replication*_j_*) + *ε_ijkl_*; where Y is the response variable, µ is the overall mean, and ε*_ijkl_* is the residual error. The treatment (trt; *i* = 1 to 6) was considered a fixed variable, and replication (replication; *j* = 1 to 2), period within replication (period; *k* = 1 to 5), and animal within replication (animal; *l* = 1 to 6) were considered random variables. Pre-planned contrasts were used to compare treatment means: PC vs. NC, RES vs. NC, NC vs. BEN, NC vs. YCW, and NC vs. MIX. The experimental unit was an individual pig. The statistical significance and tendency were determined as *p* < 0.05 and 0.05 ≤ *p* < 0.10, respectively.

## 3. Results

The analyzed DON concentration in the PC diet was 2.02 mg/kg, and the analyzed DON concentrations in the four DON-contaminated diets were 6.82, 6.35, 5.95, and 6.91 mg/kg (Table 2). During the experiment, all pigs consumed the experimental diets well and remained healthy except one pig that consumed the DON-contaminated diet supplemented with 0.5% MIX. This pig refused feed consumption during period 2, and data from this animal were not used for statistical analyses.

The average daily feed intake (ADFI) for pigs fed the uncontaminated diet (PC) was greater (*p* < 0.001; Table 3) than that for pigs fed a DON-contaminated diet with no supplemental mycotoxin-sequestering agent (NC). The average daily gain (ADG) tended to be greater (*p* = 0.093) in the PC group compared with the NC group. The gain:feed (G:F) in pigs fed the MIX diet tended to be greater (*p* = 0.064) than that in pigs fed the NC diet.

The ATTD of DM was not affected by DON contamination or the supplementation of mycotoxin-sequestering agents. However, a decrease in the ATTD of Ca (*p* = 0.032) was observed in pigs fed the YCW diet compared with the NC group. The ATTD of P in pigs fed the restricted amount of PC diet at the level of feed intake as in the NC group (RES) was less (*p* = 0.042) than that in pigs fed NC. However, the supplementation of BEN, YCW, or MIX to the NC diet did not affect the ATTD of P.

## 4. Discussion

The relatively high DON concentrations in the NC diet compared with the PC diet were anticipated due to the use of DON-contaminated barley at a 20% inclusion rate in the NC diet. The analyzed DON concentrations of the experimental diets were reasonably close to the expected values.

The reduced ADFI by DON observed in the present study is consistent with the results in previous studies [7,21,22]. Decreased feed intake is considered one of the major detrimental effects of DON in pigs. Based on a meta-analysis [7], feed intake decreases by approximately 4.4 percent when dietary DON increases by 1.0 mg/kg in pig diets. In the present study, the DON concentration in the NC diet was 4.8 mg/kg greater than that in the PC diet and the ADFI was reduced by 15%, which is reasonably close to the expected degree of ADFI reduction based on the equation suggested by Kim et al. [7]. The depressed feed intake and further depressed weight gain were likely attributed to the rapid absorption of DON in the gastrointestinal tract of pigs that could cause damages on organs. The impaired organs, including liver, spleen, and kidney atrophy, may be related to anorexia in pigs [22]. The reduced feed intake has the potential to influence the nutrient digestibility of pigs. Therefore, a treatment was included in the present study that restrictively provided the PC diet to pigs, allowing for the determination of the effects of DON itself, excluding the influence of reduced feed intake on nutrient digestibility.

Clay products, including bentonite and aluminosilicate, were reported to effectively sequester aflatoxins in in vitro studies [9,15] and to alleviate detrimental effects of aflatoxin on the growth performance of pigs [11,23]. However, clay products have shown limited effectiveness in sequestering DON [17,24], likely due to the low polarity of DON [9,15]. Yeast cell wall products composed of organic polysaccharides were reported to have beneficial effects in mitigating the harmful effects of mycotoxins [22,25,26]. However, some studies have failed to demonstrate the adsorption ability of these products against DON in in vitro experiments [9,27] or to prevent the detrimental effects of DON on the growth performance of pigs [17,24]. Enzymes, microorganisms, and preservatives such as plant extracts and antioxidants were reported to mitigate the negative effects of mycotoxins through bio-transforming and degrading mycotoxins [6,28]. Because the efficacy of mixture of these materials was unclear [24,29], more information on the DON-alleviating effects of the mixture containing enzymes, microorganisms, and plant extracts is needed. In the present study, therefore, three products of mycotoxin-sequestering agents, namely BEN, YCW, and MIX, were tested.

The lack of any effects of the supplemental mycotoxin-sequestering agents on the ADG and ADFI of pigs is consistent with previous studies that used clays [11,17,23,24], yeast products [22,24], and mixed products [24,29]. Frobose et al. [17] supplemented 0.5% montmorillonite clay product to diets contaminated with 6.4 mg/kg of DON but observed no alleviating effects on the depressed growth performance of nursery pigs caused by DON (Table 4). Similar results were observed by Thanh et al. [24], who tested supplemental aluminosilicate, a type of clay product, at a level of 0.25% and failed to alleviate the negative effects of diets containing 4.0 mg/kg of DON on the growth performance of nursery pigs. Yeast products have also been previously tested to evaluate their ability to prevent the negative effects of DON-contaminated diets on the growth performance of pigs. Among the adsorbing agents, yeast products have been found to have a relatively high affinity for DON [15], suggesting their potential to prevent DON absorption in the body of animals. The adsorbing efficacy of yeast products against DON has been observed in nonruminants [30,31,32]. However, Thanh et al. [24] reported that the supplementation of 0.1% glucomannans extracted from yeasts had no effects on the growth performance when nursery pigs consumed 4.0 mg/kg of DON in diets. Swamy et al. [22] also tested supplemental glucomannans as adsorbing agents against DON, but pigs fed diets containing wheat naturally contaminated by DON did not show improvements in growth performance. Barnes et al. [33] supplemented 0.1% yeast products to diets containing 4.0 mg/kg of DON, but failed to prevent the depressed feed intake and weight gain of nursery pigs. Based on these findings from previous experiments and the present results, the mycotoxin-sequestering agents may not be able to restore the depressed feed intake of pigs caused by dietary DON.

In contrast, the improved G:F in pigs fed the MIX-supplemented DON-contaminated diet in the present study indicates that the mixed product can partially alleviated the negative effects of DON in pigs. The mixed product used in the present study contains the live bacterium, *Eubacterium* BBSH797, which has the ability to convert DON into a non-toxic metabolite and, consequently, may improve the immune system in pigs [28]. In addition, the plant extracts contained in the mixed product may also improve immune response and cell turnover and alleviate the oxidative stress caused by mycotoxins [29]. Rather than a single product, it appears that mixed products containing a variety of detoxifying agents as well as adsorbents including clay and yeast products are relatively effective in mitigating the negative effects of DON. The supplementation of 0.15% mixed product consisting of yeast products, live bacteria, enzymes, and plant extracts to DON-contaminated diets resulted in a 13% increase in ADFI compared with the negative control group [24]. Similarly, a mixed product containing antioxidants, amino acids, microorganisms, and preservatives improved weight gain by 12% and G:F by 9% in nursery pigs compared with pigs fed diets contaminated with 4.0 mg/kg of DON with no feed additives in Barnes et al. [33]. However, the improvements in growth performance with the supplemental mixed products were not observed in other experiments [1,6,29,33,34,35]. This inconsistency might be due to differences in experimental conditions; the types of sources contaminated by DON, such as wheat, corn, and corn distillers’ dried grains with solubles as used in the literature; and the growth stages of pigs challenged by DON. However, the reason for this inconsistency still remains unclear, and therefore, further research on the mechanisms of mixed products containing a variety of detoxifying agents and adsorbent products in pigs challenged by DON is warranted.

**Table 4:** The effects of supplemental mycotoxin-sequestering agents in deoxynivalenol (DON)-contaminated diets on growth performance of pigs ^1^.

Reference	Growth Stage	Source of DON	DON in Diet (mg/kg)	Agent	Dose (%)	Experimental Period	Response
[17]	Nursery pigs	Naturally contaminated wheat	6.4	Montmorillonite	0.50	28 days	No effects on growth performance
			3.5	Montmorillonite	0.50	21 days	No effects on growth performance
[24]	Nursery pigs	Naturally contaminated wheat	4.7	Glucomannan	0.10	14 days	No effects on growth performance
			4.2	Yeast products, live bacteria, enzymes, and plant extracts	0.15	14 days	13% increase than NC group in ADFI
			4.7	Aluminosilicate	0.25	14 days	No effects on growth performance
			3.0	Blend of preservation components	0.25	14 days	60%, 14%, and 36% increase in ADG, ADFI, and G:F than NC, respectively
[22]	Nursery pigs	Naturally contaminated wheat	6.0	Glucomannan	0.20	21 days	17% decrease than NC group in ADG
[33]	Nursery pigs	Naturally contaminated corn DDGS	4.4	Yeast product	0.10	21 days	No effects on growth performance
			4.3	Yeast product and bentonite	0.15 + 0.5	21 days	No effects on growth performance
			5.1	Antioxidants, amino acids, microorganisms, and preservatives	0.25	21 days	12% increase in ADG and 9% increase in G:F than NC group
[35]	Growing pigs	Naturally contaminated wheat	2.7	Enzymes, microorganisms, and plant extracts	0.25	Over 30 days	No effects on growth performance
			3.2	Enzymes, microorganisms, and plant extracts	0.25	Over 30 days	No effects on growth performance
[34]	Nursery pigs	Naturally contaminated corn	1.2	Diatomaceous earth, kaolinite, and dehydrated yeast	0.10	28 days	No effects on growth performance
			1.2	Hydrated sodium calcium aluminosilicate	0.10	28 days	No effects on growth performance
			1.2	Hydrated sodium calcium aluminosilicate, dehydrated yeast fermentation extract, and shrimp meal	0.10	28 days	No effects on growth performance
			1.2	Sodium metabisulfite	0.30	28 days	No effects on growth performance
[6]	Nursery pigs	Naturally contaminated corn DDGS	3.2	aluminosilicates, yeast cell wall, antioxidants, prebiotics, and enzymes	0.20	5 weeks	No effects on growth performance

^1^ NC = contaminated diet with no mycotoxin-sequestering agents; ADFI = average daily feed intake; ADG = average daily gain; G:F = gain to feed ratio; DDGS = distillers’ dried grains with solubles.

In a previous study [24], supplementing a blend of preservation components containing ascorbic acid, citric acid, potassium sorbate, and sodium metabisulfite resulted in a 60% increase in weight gain, a 14% increase in feed intake, and a 36% increase in feed efficiency of nursery pigs compared with the group fed DON-contaminated diets with no additives, which are comparable to the level of a positive control. The results are partially consistent with the findings reported in Barnes et al. [33], which showed a 12% increase in weight gain and a 9% increase in feed efficiency of nursery pigs fed DON-contaminated diets supplemented with 0.25% of a mixed product including antioxidants and preservatives, although not reaching the level of a positive control. Thanh et al. [24] suggested that the improvements in growth performance observed in pigs subjected to the blend of preservation components may be attributed to the lower analyzed DON concentration in the contaminated diets, approximately 3.0 mg/kg of DON, compared with other treatments with 4.0 mg/kg of DON. However, as indicated in Table 4, depressed growth performance caused by DON concentrations in diets less than 3.0 mg/kg was not restored by supplementing other mycotoxin-sequestering agents including clay and yeast products in other studies. Further research is warranted to clearly verify the complex mechanisms of preservatives and antioxidants that mitigate the native effects on the growth performance of pigs.

The effects of feeding DON-contaminated diets to pigs on DM digestibility have been inconsistent among previous studies. Kong et al. [8] and Goyarts et al. [36] reported that feeding DON-contaminated diets positively affected DM digestibility compared with the control group. This may be partially explained by the increased activity of protease and carbohydrase resulting from the consumption of mycotoxin-contaminated grains [37]. In contrast, Thanh et al. [24] and Holanda et al. [38] observed the lower DM digestibility in weanling pigs fed DON-contaminated diets compared with the control group. The lack of changes in DM digestibility due to DON in the present study is consistent with the findings by Dänicke et al. [1] and Jo et al. [5]. Although the reason for inconsistent responses in the DM digestibility by DON is unclear, growth stages of pigs may be one of the factors that influence the digestibility responses by DON. Dietary DON lowered DM digestibility in nursery pigs [24,38] but not in growing pigs [1,5] likely due to the less susceptibility to DON in older pigs.

In the present study, the lack of positive effects of supplemental mycotoxin-sequestering agents on DM digestibility of pigs is likely due to the absence of depressed DM digestibility caused by DON contamination. The present observations are in agreement with Dänicke et al. [35] who observed no improvement in DM digestibility of pigs by adding a mixed product to DON-contaminated diets (Table 5). In contrast, Thanh et al. [24] reported increased DM digestibility by supplementation of glucomannans or a mixed product to DON-contaminated diets.

The reduced ATTD of Ca in pigs fed the YCW diet compared with those fed the NC diet in the present study was not anticipated. Previous studies have consistently reported an increased ATTD of Ca in pigs affected by dietary DON, regardless of the supplementation of mycotoxin-sequestering agents [24,39]. According to the studies that investigated the blood characteristics in pigs associated with feeding DON-contaminated diets, decreases in the serum Ca concentration of the pigs were observed [40,41]. These findings suggest that pigs with low serum Ca may have stimulated intestinal absorption and increased utilization of Ca in their diets [42]. However, the reason for the reduced Ca digestibility observed with the supplemental YCW in pigs fed a DON-contaminated diet remains unclear.

The greater utilization of P in pigs fed DON-contaminated diets compared with the RES group is consistent with the results from a previous study [39] that fed a diet containing 3.6 mg DON/kg to growing pigs. In addition, Thanh et al. [24] also reported a higher retention-to-intake ratio of P in pigs fed DON-contaminated diets containing approximately 4 mg DON/kg, regardless of anti-mycotoxin additive supplementation. Thanh et al. [24] reported no changes in the P digestibility of pigs after supplementation with glucomannan or aluminosilicate, which is consistent with the present results as well. Additionally, in the present study, supplementing the mixed product did not cause a change in the P digestibility of pigs. This finding aligns with the observation that supplementing a mixed product containing yeast products, live bacteria, enzymes, and plant extracts in the previous study resulted in a 3% increase in P digestibility compared with pigs fed DON-contaminated diets with no additives.

## 5. Conclusions

The supplementation of mycotoxin-sequestering agents could not compensate for reduced feed intake and growth performance, but a mixed product consisting of enzymes, microorganisms, minerals, and plant extracts tended to improve the feed efficiency of pigs. Mycotoxin-sequestering agents did not affect nutrient utilization in deoxynivalenol-contaminated diets, except for yeast cell wall products for calcium digestibility. Based on the present work, the use of a mixed product consisting of enzymes, microorganisms, minerals, and plant extracts is suggested, and the reduction in calcium digestibility by yeast cell wall products needs to be considered in diet formulations. In previous studies, most single mycotoxin-sequestering agents showed no effects in compensating for reduced growth performance or nutrient digestibility in pigs. However, mixed products containing various agents, plant extracts, or antioxidants often had a positive effect on the growth and nutrient digestibility in pigs. This suggests that these products might contribute to improved pig health and at least partially compensate for the reduced performance caused by dietary deoxynivalenol.

## Figures and Tables

**Table 1:** Concentrations of deoxynivalenol in two sources of barley (as-fed basis).

Item	Contaminated Barley	Uncontaminated Barley
Deoxynivalenol, mg/kg	26.0	1.0

**Table 2:** Ingredient composition and chemical composition of experimental diets (as-fed basis) ^1^.

Items	PC	NC	Bentonite	Yeast Cell Wall	MIX
Ingredient, %					
Ground corn	52.90	52.90	52.90	52.90	52.90
Soybean meal, 48% crude protein	22.00	22.00	22.00	22.00	22.00
Uncontaminated barley	20.00	-	-	-	-
Contaminated barley ^2^	-	20.00	20.00	20.00	20.00
Soybean oil	2.00	2.00	2.00	2.00	2.00
l-Lys·HCl, 78.8%	0.20	0.20	0.20	0.20	0.20
Dicalcium phosphate	0.80	0.80	0.80	0.80	0.80
Ground limestone	0.80	0.80	0.80	0.80	0.80
Vitamin-mineral premix ^3^	0.40	0.40	0.40	0.40	0.40
Salt	0.40	0.40	0.40	0.40	0.40
Corn starch	0.50	0.50	-	-	-
Mycotoxin-sequestering agent	-	-	0.50	0.50	0.50
Analyzed chemical composition					
Dry matter, %	90.46	90.61	89.93	89.38	90.17
Gross energy, kcal/kg	4164	4043	4103	4099	4069
Calcium, %	0.90	1.23	1.06	1.18	1.13
Phosphorus, %	0.48	0.51	0.49	0.52	0.50
Deoxynivalenol, mg/kg					
Calculated	1.89	6.89	6.89	6.89	6.89
Analyzed	2.02	6.82	6.35	5.95	6.91

^1^ PC = uncontaminated diet; NC = contaminated diet; MIX = NC + a mixture product consisting of enzymes, microorganisms, minerals, and plant extracts. ^2^ The contaminated barley contained 26.0 mg/kg of deoxynivalenol. ^3^ Provided the following quantities per kg of complete diet: vitamin A, 12,500 IU; vitamin D_3_, 1000 IU; vitamin E, 125 IU; vitamin K_3_, 6.3 mg; thiamin, 6.3 mg; riboflavin, 25.0 mg; pyridoxine, 12.5 mg; vitamin B_12_, 0.1 mg; pantothenic acid, 100 mg; folic acid, 7.5 mg; niacin, 225 mg; biotin, 0.5 mg; Cu, 87.5 mg as copper sulfate; Fe, 125 mg as iron sulfate; I, 1.0 mg as potassium iodate; Mn, 75 mg as manganese sulfate; Se, 0.25 mg as sodium selenite; and Zn, 60 mg as zinc oxide.

**Table 3:** Effects of mycotoxin-sequestering agent in deoxynivalenol-contaminated diets on growth performance and apparent total tract digestibility (ATTD) of nutrients ^1^.

Items	Uncontaminated		Contaminated				SEM	*p*-Value for Contrast				
	PC	RES	NC	BEN	YCW	MIX		PC vs. NC	RES vs. NC	NC vs. BEN	NC vs. YCW	NC vs. MIX
No. of observations	9	9	9	9	9	8						
Growth performance ^2^												
Daily feed intake, kg/d	2.77	2.35	2.35	2.35	2.36	2.26	0.17	<0.001	0.255	0.937	0.932	0.255
Average daily gain, kg/d	0.811	0.758	0.714	0.675	0.694	0.799	0.057	0.093	0.434	0.501	0.733	0.155
Gain:feed	0.305	0.331	0.314	0.297	0.304	0.357	0.032	0.682	0.438	0.447	0.649	0.064
Digestibility, %												
ATTD of dry matter	87.1	86.8	86.2	86.0	85.8	86.6	0.5	0.176	0.318	0.778	0.529	0.550
ATTD of Ca	65.9	65.0	64.7	66.3	60.6	65.0	2.4	0.516	0.890	0.397	0.032	0.900
ATTD of P	55.7	52.7	57.2	57.9	57.8	57.6	2.0	0.484	0.042	0.755	0.791	0.846

^1^ PC = uncontaminated diet; RES = restricted feeding of an uncontaminated diet at the level of feed intake as in the NC group; NC = deoxynivalenol-contaminated diet; BEN = NC + 0.5% bentonite; YCW = NC + 0.5% yeast cell wall; MIX = NC + 0.5% mixture product consisting of enzymes, microorganisms, minerals, and plant extracts. All pigs except the RES groups had ad libitum access to the diets. ^2^ Growth performance was measured using a replicated 6 × 5 incomplete Latin square design with 12 pigs and 5 periods. The growth performance data for one replication were obtained from a period of 12 days. During the last period, only 6 animals were used due to the lack of experimental diets, resulting in 9 observations for each treatment group.

**Table 5:** The effects of supplemental mycotoxin-sequestering agents in deoxynivalenol (DON)-contaminated diets on nutrient digestibility of pigs ^1^.

Reference	Growth Stage	Source of DON	DON in Diet (mg/kg)	Agent	Dose (%)	Response
[35]	Finishing pigs	Naturally contaminated wheat	3.2	Enzymes, microorganisms, and plant extracts	0.25	No effects on organic matter digestibility
[39]	Growing pigs	Naturally contaminated barley	4.0	Sodium meta-bisulfite and antioxidant blend	0.30	No effects on DM and Ca digestibility and 12% decreased in P digestibility than NC
[24]	Nursery pigs	Naturally contaminated wheat	4.7	Glucomannan	0.1	3% increase in DM digestibility than NC and no effects on Ca and P digestibility
			4.2	Yeast products, live bacteria, enzymes, and plant extracts	0.15	3% increase in DM digestibility, 5% increase in P digestibility, and no effects on Ca digestibility
			4.7	Aluminosilicate	0.25	No effects on DM, Ca, and P digestibility
			3.0	Blend of preservation components	0.25	12% decrease in P digestibility and no effects on DM and Ca digestibility

^1^ DM = dry matter; NC = contaminated diet with no mycotoxin-sequestering agents.

## Data Availability

The data presented in this work are available.
